# Immune Dysregulation in Patients With Chromosome 18q Deletions—Searching for Putative Loci for Autoimmunity and Immunodeficiency

**DOI:** 10.3389/fimmu.2021.742834

**Published:** 2021-11-17

**Authors:** Anna Hogendorf, Maciej Zieliński, Maria Constantinou, Robert Śmigiel, Jolanta Wierzba, Krystyna Wyka, Anna Wędrychowicz, Anna Jakubiuk-Tomaszuk, Edyta Budzynska, Malgorzata Piotrowicz, Beata S. Lipska-Ziętkiewicz, Ewa Kaczorowska, Agata Cieślikowska, Anna Kutkowska-Kaźmierczak, Jolanta Fijak-Moskal, Monika Kugaudo, Małgorzata Kosińska-Urbańska, Agnieszka Szadkowska, Maciej Borowiec, Maciej Niedźwiecki, Piotr Trzonkowski, Wojciech Młynarski

**Affiliations:** ^1^ Department of Pediatrics, Diabetology, Endocrinology and Nephrology, Medical University of Lodz, Lodz, Poland; ^2^ Department of Medical Immunology, Medical University of Gdansk, Gdansk, Poland; ^3^ Department of Clinical Genetics, Medical University of Lodz, Lodz, Poland; ^4^ Department of Pediatrics, Division of Pediatrics and Rare Disorders, Wroclaw Medical University, Warsaw, Poland; ^5^ Department of Internal and Pediatric Nursing, Medical University of Gdansk, Gdansk, Poland; ^6^ Department of Pediatrics, Oncology and Hematology, Medical University of Lodz, Lodz, Poland; ^7^ Polish-American Pediatric Institute, Jagiellonian University Collegium Medicum, Department of Pediatric and Adolescent Endocrinology, Cracow, Poland; ^8^ Department of Pediatric Neurology and Rehabilitation, Medical University of Bialystok, Białystok, Poland; ^9^ Department of Genetics, Polish Mother’s Memorial Hospital—Research Institute, Lodz, Poland; ^10^ Clinical Genetics Unit, Department of Biology and Medical Genetics, Medical University of Gdansk, Gdansk, Poland; ^11^ Department of Biology and Medical Genetics, Medical University of Gdansk, Gdansk, Poland; ^12^ Department of Medical Genetics, Children’s Memorial Health Institute, Warsaw, Poland; ^13^ Department of Medical Genetics, Institute of Mother and Child, Warsaw, Poland; ^14^ Outpatient Genetic Clinic, University Children’s Hospital of Cracow, Cracow, Poland; ^15^ Department of Children and Adolescent Psychiatry, University Clinical Center, Pediatric Teaching Clinical Hospital Warsaw, Warsaw, Poland; ^16^ Department of Pediatrics, University of Zielona Gora, Zielona Gora, Poland; ^17^ Department of Pediatrics, Hematology and Oncology, Medical University of Gdansk, Gdansk, Poland

**Keywords:** 18q deletion syndrome, immune deficiency, type 1 diabetes, autoimmune diseases, thyroiditis, T regulatory cells, antibody deficiency

## Abstract

**Introduction:**

Autoimmune disorders, IgA deficiency, and allergies seem to be common among individuals with 18q deletion syndrome [OMIM 601808]. We aimed to determine the prevalence, mechanism, and genetic background of autoimmunity, immune deficiency, and allergy in a cohort of patients with 18q deletions.

**Material and Methods:**

Medical registries and social media were used to recruit the patients. Microarray oligonucleotide comparative genomic hybridization (aCGH) (Agilent, Santa Clara, CA, USA) was performed in all patients to identify size and location of chromosome 18 deletion. Clinical evaluation and medical record collection were performed in each of the study participants. The history of autoimmune disorders, severe and/or recurrent infections, and symptoms of allergy were noted. Total immunoglobulin IgG, IgA, IgM, IgE, and IgG_1-4_ serum levels were measured using nephelometry and ELISA methods. Lymphocyte T subset phenotyping was performed in 24 subjects from 18q del cohort. To predict the most promising candidate genes, we used the ENDEAVOUR—a free web resource for gene prioritization.

**Results:**

18q deletion was confirmed by means of array CGH analysis in 27 individuals, 15 (55.6%) females and 12 males, referred to the project by specialists in medical genetics, diabetology, or pediatric endocrinology between May 2015 and December 2019. The mean age at examination was 11.8 years (min–max: 4.0–33.5). Autoimmune disorders were present in 14/27 (51.8%) of the cohort. In eight of patients, symptoms of immune deficiency coexisted with autoimmunity. Allergy was reported in nine of 27 (33.4%) patients. Over 89% of patients presented with at list one type of immunoglobulin (IgA, IgM, IgG, IgE, and IgG_1-4_) deficiency and eight of 25 (32%) had abnormalities in at least two major immunoglobulin (IgG, IgA, IgM) measurements (CVID-like phenotype). Patients with 18q del exhibited a significantly decreased CD4, Treg FOXP3+, TregFOXP3+Helios+, and TemCD4 cell numbers in comparison with the control groups of 24 T1DM patients and 28 healthy controls.

**Conclusions:**

Patients with 18q deletions frequently suffer from autoimmune disorders, recurrent infections, and allergy due to immune dysregulation presenting with variable antibody deficiencies and T-regulatory cell deficiency (CD4+CD25+CD127lowFOXP3+). The spectrum of speculations regarding which gene might be responsible for such phenotype ranges from single gene haploinsufficiency to deletion of a cluster of immunogenes located distally to 18q21.

## Introduction

Chromosome 18q deletion syndrome (de Grouchy syndrome, OMIM 601808) results from a loss of a fragment of the long (q) arm of 18 chromosomes. It was first described in 1964 (1) and occurs in about one in 40,000–55,000 live births (2). The syndrome is known to have a heterogeneous presentation that includes multiple congenital anomalies such as short stature, developmental delay, and intellectual disability, hypotonia, microcephaly, and other dysmorphic features such as hypoplasia of the midsection of the face with wide carp-like mouth, as well as skeletal deformities such as hand and foot deformities, genital anomalies, vision, and hearing impairment. Less common signs include cleft lip and palate, congenital heart disease, hypertelorism, clinodactyly, umbilical and inguinal hernia, convulsions, and feeding problems (3, 4). Most 18q deletions are terminal and localized in the distal half of the long arm (18q21.1-qter). However, smaller interstitial deletions, residing in the region between q12 and q21 and associated with a milder phenotype have also been described (5). About 94% of cases with 18q deletion syndrome appearance are *de novo*, and the remaining 6% are the inherited from a parent carrying a balanced chromosomal translocation.

We have previously reported on a girl with 18q del syndrome, type 1 diabetes mellitus, autoimmune thyroiditis, vitiligo, and recurrent infections due to immunoglobulin A, E, and G_4_ deficiency. She was also found to have CD3+CD4+FoxP3+, CD3+CD4+FoxP3+CD25+, and CD3+CD4+CD25+CD127+ regulatory T cell deficiency. Therefore, we hypothesized that the exceptional coincidence of the three autoimmune disorders occurring at an early age and immune deficiency is associated with the deletion of certain gene(s) located on deleted region at 18q21.32-q23 (chr18:58,660,699-78,012,870) (6).

Our hypothesis is strongly supported in literature by several case reports on the coexistence of autoimmune thyroiditis, pernicious anemia, vitiligo, and also immunoglobulin (Ig)A deficiency and CVID in patients with the syndrome (7–9).

This study was therefore undertaken to evaluate the prevalence of autoimmune disorders, recurrent/severe infections, and allergies among patients with 18q del syndrome and to check if the previously observed hypogammaglobulinemia and TregFOXP3+ deficiency are common features in these patients. Additional aim was to identify molecular background of the immune dysregulation by prioritization of deleted genes.

## Methods

### Patients and Samples

The study was approved by the Ethics Committee of the Medical University of Lodz. The information about the project was spread among stakeholders at the Departments of Medical Genetics and Pediatric Endocrinology countrywide, The Polish Registry of Congenital Defects, the Polish Association for Rare Diseases (patient society), Diabetes Poland (medical association) *via* internet and during Medical Conferences and Symposia. Social media, e.g., Facebook, Rare Connect were also helpful in the patients’ recruitment. Patients were recruited in collaboration with 12 medical genetics or endocrinology centers and cytogenetic laboratories from eight major Polish cities (Lodz, Warsaw, Krakow, Gdansk, Poznan, Wroclaw, Zielona Gora, and Bialystok). The deletions were previously diagnosed by standard cytogenetic methods. The written informed consent was obtained from each study subject and/or legal guardian in accordance with the Declaration of Helsinki.

The participants were evaluated on-site in the participating centers by our team of investigators; some of them several times. Data on medical history, especially concerning frequent and recurrent infections, autoimmune and nonautoimmune comorbidities and detailed family history, as well as laboratory results were obtained from the children’s parents and from all relevant medical records.

At the same time, peripheral venous blood samples were obtained, but only from those who were otherwise healthy.

For the purpose of the T-regulatory cells, assessment peripheral venous blood samples were also obtained from two age and sex-matched control groups—24 patients with autoimmune diabetes (T1D) and 28 healthy controls.

### Samples Collection

Peripheral venous blood drawn into 2.9 ml EDTA tubes was sent to the Department of Clinical Genetics, Medical University of Lodz for genetic evaluation (aCGH).

Serum samples (4 ml) were prepared by centrifugation at 3,000×*g* for 10 min, aliquoted, and sent to the “APC” laboratory until IgA, IgM, IgG, IgE, and IgG_1_-IgG_4_ level assessment and to study center of the Department of Pediatrics, Oncology and Hematology, Medical University of Lodz, where they were stored at −80°C for future analyses.

Peripheral venous blood (4–6 ml) for immunophenotyping was drawn into anticoagulanted EDTA tubes and sent overnight in styrofoam cooler boxes (not frozen) to the Department of Clinical Immunology and Transplantology, Medical University of Gdansk, Poland.

### Array Comparative Genome Hybridization

DNA was isolated from peripheral blood by DNA isolation kit (Qiagen, www.qiagen.com). Array comparative genome hybridization (aCGH) was performed by an Agilent Human Genome SurePrint G3 CGH ISCA v2 Microarray Kit, 8x60K (Agilent, www.agilent.com). A 60-mer oligonucleotyde-based microarray (60 k aCGH) with 18,851 probes in ISCA regions and 40,208 backbone probes was used, which allows for genome-wide survey and molecular aberration typing with resolution of approximately 180 kb. A 60-k array allows for detection of copy number variations (CNVs) larger than 500 kb. DNA—both the control one and that from the patient, was labeled using Klenov’s method, following the protocol of Agilent. A volume of 200 ng of purified patient’s DNA and DNA from a male control was double digested with restriction enzyme AluI and RSAI (Promega, www.promega.com) at 37°C for 2 h. Following purification on columns, 1 µg of digested patient’s DNA and of the male control’s DNA were labeled by Cy5-dUTP and Cy3-dUYP, respectively, using the random priming method (Invitrogen, www.invitrogen.com) at 37°C for 2 h. The labeled DNA was purified on the column, mixed, denaturated, and prehybridized with 50 µg of Cot-1 DNA at 37°C for 30 min, then applied onto the array and hybridized at 65°C in a hybridization stove with rotation for 40 h. After hybridization, the array was twice washed in aCGH wash buffers 1 and 2 and then analyzed by an INOPSYS 900 AL scanner and the Feature Extraction for CytoGenomics program. Genomic analysis and graphic presentation of the results was generated by the Agilent CytoGenomics software package (Edition 2.9.2.4).

### Immunoglobulin Assays

Total immunoglobulin IgG, IgA, IgM, and IgE serum levels were measured in all the patients in a reference “APC” laboratory (supported by Labquality and Cequal external quality assessment). IgG, IgA, IgM, and IgE concentrations were measured with nephelometry using a Binding Site, Birmingham, UK according to the manufacturer’s instructions. IgG subclasses IgG1, IgG2, IgG3, or IgG4 levels were determined in patients with IgGD and/or IgAD using PeliClass human IgG subclass ELISA kit M1551, Sanquin Reagents. Samples with an IgE concentration below the lower limit of detection of the nephelometric IgE assay were reevaluated by the total IgE low-range enzyme-linked fluorescent assay on a Mini Vidas instrument (bioMérieux S.A., Marcy l’Étoile, France) to obtain a precise determination.

As immunoglobulin levels in the pediatric population are age dependent, for the purpose of the study, age-adjusted reference values for IgA, IgM, IgG, and IgG_1-4_ levels, determined in healthy children in Mazovia district, were used (10).

### Definitions

Autoimmune diseases—diseases occurring when the immune system attacks self-molecules as a result of a breakdown of immunologic tolerance to autoreactive immune cells strongly associated with genetic, infectious, and/or environmental predisposing factors (e.g., autoimmune thyroiditis, type 1 diabetes, celiac disease, juvenile idiopathic arthritis, immune thrombocytopenic purpura, autoimmune neutropenia, ulcerative colitis, Crohn’s disease).

Severe infections—infections requiring hospitalization to administer specific treatment, not possible in-home care (intravenous antibiotic treatment, oxygen therapy, resuscitation).

Allergy—an unnecessary immune response to an innocuous substance (an allergen). Examples of common substances people are allergic to include pollens, dust mites, insect venom, and food such as nuts or shellfish. Allergic reactions can be grouped into two classes. The most common and best understood is mediated by a class of antibody called IgE. Other reactions are non-IgE mediated and typically cause symptoms to appear more slowly, sometimes several hours after exposure. In fact, non-IgE-mediated reactions do not necessarily involve antibodies but instead, cell reactions of the immune system. They are much less common and are generally less well-understood. The most common way to diagnose an IgE-mediated allergy is through a blood test to identify allergen-specific IgE or a skin prick test which results in a local inflammatory reaction after administration of the trigger allergen. Allergy may manifest as eczema, allergic rhinitis and conjunctivitis, food allergy, asthma, urticaria, angioedema, and anaphylactic shock.

Anaphylaxis is a serious, life-threatening, generalized hypersensitivity reaction that can occur *via* immunologic (either IgE-dependent or IgE-independent) or nonimmunologic mechanisms (11).

According to the diagnostic criteria for PID established by the European Society for Immunodeficiencies (ESID), selective IgAD deficiency was defined by serum levels of IgA less than 0.07 g/l in the presence of normal IgG and IgM in a patient older than 4 years, in whom other causes of hypogammaglobulinemia have been excluded (12). IgG and IgM deficiency was considered when it was below age-adjusted reference values (12). Selective IgE deficiency (IgED) was defined as a significant decrease in serum levels of IgE (<2 kIU/L) in the presence of normal IgG and IgM (13). Complex hypogammaglobulinemia or CVID-like phenotype—was defined by reduced total serum IgG and IgA and/or IgM levels. The definitions of immunoglobulin deficiencies are given in **Table 1**.

**Table 1:** Immunoglobulin deficiency categories considered in the study^a^.

	Abbreviation	Total IgA	Total IgG	Total IgM	Total IgE
Immunoglobulin A deficiency	IgAD	2 SD below the mean for given age^a^	NC	NC	NC
Selective immunoglobulin A deficiency	sIgAD	<0.07 g/l^a^	WNL	WNL	NC
Immunoglobulin G deficiency	IgGD	NC	<Age-adjusted reference	NC	NC
Selective immunoglobulin G deficiency	sIgGD	WNL	<Age-adjusted reference	WNL	NC
Immunoglobulin M deficiency	IgMD	NC	NC	<Age-adjusted reference	NC
Selective immunoglobulin M deficiency	sIgMD	WNL	WNL	<Age-adjusted reference	NC
Immunoglobulin E deficiency	IgED	NC	NC	NC	<2 kIU/l
Selective immunoglobulin E deficiency	sIgED	WNL	WNL	WNL	<2 kIU/l
CVID-like immunoglobulin deficiency	CVID-like IgD	≥2 immunoglobulin class deficiencies (IgA and/or IgG and/or IgM)			NC

a All study subjects were older than 4 years of age and other causes of hypogammaglobulinemia have been excluded.

SD, standard deviation; NC, not considered for classification; WNL, within normal limits.

### Lymphocyte Subset Phenotyping

Regulatory T cells were tested using EDTA whole blood samples. First, PBMC was isolated with a density gradient method, Ficoll Paque Plus (GE Healthcare, Chicago, IL, USA). Samples were checked for viability and 80% cutoff was applied, as tested with trypan blue method. Then 250,000 cells were stained with CD3 V500 (clone UCHT1), CD4 PerCP (SK3), CD25 PE (clone 2A3), CD127 FITC (clone HIL-7R-M21), CD45RA PE-Cy7 (clone L48) (all from BD Biosciences, San Jose, CA, USA), and CD62L APC eFluor 780 (clone DREG56) (Thermo Fisher Scientific, Waltham, MA, USA) monoclonal antibodies and permeabilized with Foxp3 Transcription Factor Staining Buffer Set (Thermo Fisher Scientific, Waltham, MA, USA). Next, cells were stained with FoxP3 APC (clone), Helios eFluor 450 (clone 22F6) (all from Thermo Fisher Scientific, Waltham, MA, USA), and finally readout with FACS CANTOII equipped with FACS Diva software flow cytometer (BD Biosciences, San Jose, CA, USA). For data analysis, fluorescence minus one (FMO) approach was used to set gates adequately. First, doublets were excluded according to FSC A/H signal distribution, then CD3 high expressing lymphocytes were gated as CD4/CD3 double-positive T cells. Next, CD4+ T cells were gated and Tregs were identified as either CD127low/CD25+ or CD25/FoxP3 cells. Finally, Helios expression was checked in Tregs population and naïve/memory CD4 T lymphocytes were analyzed according to CD62L/CD45RA expression. For every sample, a minimum of 100,000 events were collected.

### Gene Prioritization

To predict the most promising candidate genes, we used the *ENDEAVOUR*—a free web resource for gene prioritization (https://endeavour.esat.kuleuven.be). Shortly, the strategy is based on how similar a candidate gene is to a profile derived from genes already known to be involved in the process of interest. The approach relies on the integration of multiple heterogeneous sources (e.g., coding sequence, gene expression, functional annotation, literature, regulatory information) that cover what we currently know about these genes (14). In the first step, we selected 20 genes which are widely known to be associated with autoimmunity and/or immunodeficiency (*STAT1*, *STAT3*, *IL2RA*, *PIK3R1*, *CTLA4*, *FASLG*, *TYK2*, *CD19*, *FOXP3+*, *TNFRSF13B*, *PIK3CD*, *CD81*, *ICOS*, *CR2*, *ITCH*, *AICDA*, *AIRE*, *MS4A7*, *LRBA*). These genes severed as a training set. In the second step, we selected the whole long arm of chromosome 18 with 284 identified genes for prioritization with the *ENDEAVOUR* approach. We performed four separate iterations, choosing sequence data, expression data, functional annotations (protein-protein interaction networks text mining, regulation information), and phenotypic information as the data source. Finally, the rankings (one per data source) were fused into a global ranking, using Order Statistics. We then selected top 10 genes that were ranked at the top in each iteration.

Having the top 10 candidates, we performed statistical analysis to study the association between the deletion of each candidate gene and the presence of disturbances within each CD4 T-cell compartment.

### Statistical Analysis

The Statistica 12.5 PL package (Statsoft, Tulsa, OK, USA) was used for the analysis. Continuous variables are presented as medians followed by interquartile ranges (IQR), while nominal variables are presented as numbers followed by percentages in brackets. The Shapiro-Wilk test was used to assess the normality of distribution. Continuous variables were compared using the Mann-Whitney *U*-test, Wilcoxon, and Kruskal-Wallis ANOVA in the case of a nonnormal distribution or *t*-test in case of normal distribution. *p*-values <0.05 were considered statistically significant.

## Results

Thirty-six patients were referred to the project by specialists in medical genetics, diabetology, or pediatric endocrinology between May 2015 and December 2019.

18q deletion was confirmed by array CGH analysis in 27 of them, 15 (55.6%) females and 12 males. The mean age at examination was 11.8 years (min–max: 4.0–33.5). Clinical characteristics are given in **Table 2**, and the particular phenotypic features are listed in **Table 3**.

**Table 2:** Clinical characteristics of patients with 18q del syndrome.

Clinical feature	18qdel (*n* = 27)	T1D (*n* = 24)	Healthy controls (*n* = 24)
Age (years)*	11.9 (4.0–33.5)	12.3 (4.0–18)	11.6 (4.2–18)
**Gender (F/M)**	15/12	13/11	12/12
**18q deletion type**			
Terminal	24/27 (88.9%) (including 3 cases with ring 18)	NA	NA
Interstitial	3/27 (11.2%)		
Ring 18	3/27 (11.2%)		
*De novo*	22/27 (81.5%)		
Mosaic	2/27 (7.0%)		
**Autoimmune comorbidities**	**14/27 (51.8%)**	**4 (16.7%)**	**0**
Autoimmune thyroiditis	14/27 (51.8%)	3 (12.5%)	
Hashimoto thyroiditis	13/27 (48.1%)		
Grave’s disease	1/27 (3.7%)		
Type 1 diabetes	2/27 (7.4%)		
Vitiligo	1/27 (3.7%)	1 (4.1%)	
Alopecia areata	3/27 (11.2%)		
Autoimmune thrombocytopenia	1/27 (3.7%)		
**Allergy**	**9/27 (33.4%)**	**6 (25%)**	**0**
Allergic rhinitis and conjunctivitis	0/27 (0%)	5 (20.8%)	
Eczema	6/27 (22.3%)	1 (4.1%)	
Asthma	0/27 (0%)	1 (4.1%)	
Food allergy	4/27 (%)		
Anaphylaxis	1/27 (3.7%)		
Urticaria	1/27 (3.7%)		
**Symptoms of immunodeficiency**	**13/27 (48.1%)**	**0**	**0**
Recurrent respiratory tract infections	10/27 (37%)		
Recurrent urinary tract infections	5/27 (18.5%)		
Recurrent gastrointestinal tract infections	5/27 (18.5%)		
Sepsis	3/27 (11.2%)		
**Immunoglobulin deficiency**	**22/25 (88%)**		
IgAD	5/25 (20%)		
sIgAD	3/25 (12%)		
IgMD	10/25 (40%)		
sIgMD	2/25 (8%)		
IgGD	8/25 (32%)		
IgED	13/25 (52%)		
sIgED	6/25 (24%)		
CVID-like phenotype	8/25 (32%)		
IgG subclass deficiency	8/19(42.1%)		
IgG_1_	2/19 (10.5%)		
IgG_2_	0/20 (0%)		
IgG_3_	2/23 (8.6%)		
IgG_4_	5/23 (21.7%)		

*p-value > 0.05. NA, not applicable.

**Table 3:** Phenotypic features of 27 patients with 18q deletion.

Phenotypic feature	*n* (%)
Developmental delay	26/27 (96.2%)
Mental retardation	25/27 (92.6%)
Facial dysmorphism	26/27 (96.3%)
Hypotonia	24/27 (89%)
Motor clumsiness/dyscoordination	23/27 (85.2%)
Foot or hand deformity	23/27 (85%)
Midface hypoplasia	21/27 (77.8%)
Short/flat philtrum	20/27 (74%)
Downturned corners of mouth	20/27 (74%)
Flat nasal bridge	20/27(74%)
Hearing loss/deafness	18/25 (72%)
Dysplastic ears	18/27 (66.7%)
High or cleft lip/palate	18/27 (66.7%)
Delayed myelination (MRI)	10/15 (66.7%)
Hyperextensible joints	17/27 (63%)
Microcephaly	16/27 (59.2%)
Narrow auditory canals	16/27 (59.2%)
Up/down slanting palpebral fissures	16/27 (59.2%)
Microphthalmia/head circumference	15/27 (55.6%)
Decreased growth	15/27 (55.6%)
Prognathism	14/27 (51.8%)
Congenital heart disease	13/27 (48%)
Reproductive organ defects (hypoplasia, cryptorchid testes, hypospadiasis, micropenis)	8/27 (29.6%)
Reproductive organ defects (hypoplasia, cryptorchid testes, hypospadiasis, micropenis)	8/27 (29.6%)
Strabismus	8/27 (29.6%)
Tremor	8/27 (29.6%)
Autism	7/27 (26%)
Seizures	6/27 (22.3%)
Nystagmus	6/27 (22.3%)
Umbilical hernia	5/27 (18.5%)
Urinary tract malformation	5/27 (18.5%)
Aural atresia	1/27 (3.7%)

**Figure 1:**
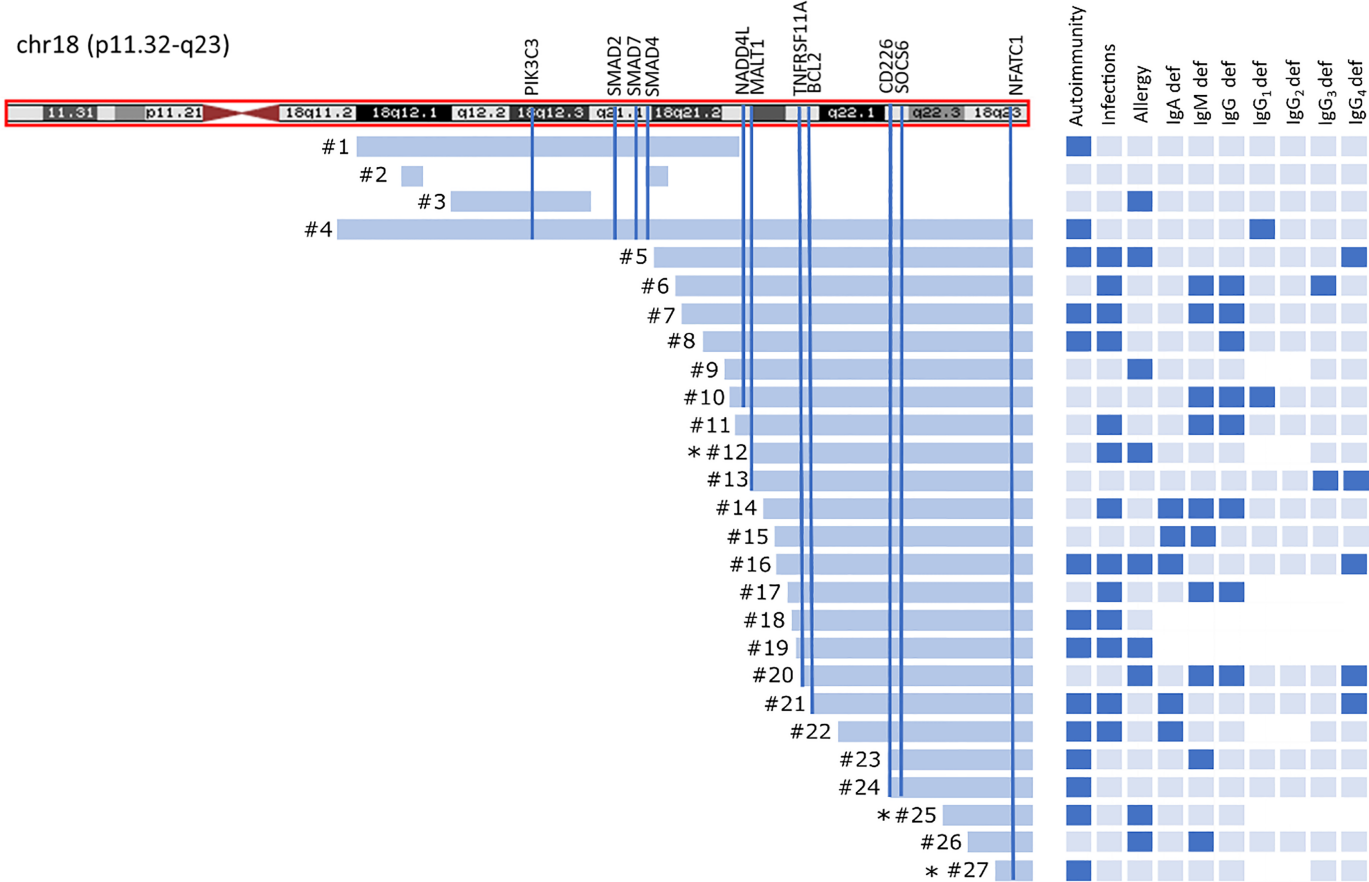
Chromosome 18 ideogram showing the mapping of breakpoints in 18qdel cohort. Light blue bars depict deleted fragments of q arm each of the 27 study subjects in relation to clinical and laboratory findings. Black asterisks indicates ring chromosome 18 with simultaneous deletion at 18p.

Twenty-four patients had “pure” 18q deletion and three of the patients had a ring chromosome 18 (deletion from both p and q arms). The majority of the patients 23/27 (85.2%) presented with the so-called distal 18q del syndrome due to a deletion distally to 18q21.1 (from 46,700,000 to the end of the chromosome at 78,077,248 bp *hg 19 nucleotide scale that includes 103 genes). One patient had proximal 18q del, one had both proximal and distal interstitial deletion, one proximal terminal deletion encompassing almost the whole arm of 18q (25% mosaic), and one proximal encompassing a little fragment distally to 18q21.1. The length of the q arm deletion varied from very small interstitial deletions encompassing 1,007 Mbp to almost the whole arm of 18q (55.00 Mbp). The deleted q fragments are depicted on the chromosome ideogram (**Figure 1**) and detailed microarray analysis results of each patient are given in the **Supplementary Table S1**. Two patients had a mosaic genotype. In 22/27(81.5%) of cases, aberration occurred *de novo.*

Autoimmune disorders were present in 14/27 (51.8%) of the cohort. Autoimmune thyroiditis was found in 14/27 (51.8%), alopecia areata in three of 27(11.2%), type 1 diabetes in two of 27 (7.4%), vitiligo in one of 27 (3.7%), and autoimmune thrombocytopenia in one of 27(3.7%).

Thirteen of 27 (48.1%) of the patients suffered from severe/recurrent infections. In eight of them, the symptoms of immune deficiency coexisted with autoimmunity. Recurrent respiratory tract infections at 10/27 (37%), followed by urinary tract infections and gastrointestinal tract infections (both in 5/27 (18.5%) were the most commonly reported. Sepsis was reported in three cases (11.2%). Only three patients had been initially diagnosed with immunodeficiency and had been referred to Clinical Immunology Outpatient Clinic. Allergy was reported in nine of 27 (33.4%) patients, including food allergy at four of 27 (14.8%), eczema at six of 27 (22.3%), urticaria at one of 27 (3.7%), and anaphylaxis at one of 27(3.7%). Autoimmunity, infections, and allergy coexisted in three of 27 cases, autoimmunity and allergy at one of 27, and autoimmunity and infections at five of 27 patients (**Figure 1**).

### Immunoglobulins A, M, G, E, and IgG Subclass Levels

Immunoglobulins A, G, M, E, and IgG subclasses were measured in nearly all patients, with a few exceptions (**Figure 1**).

Seventeen of 19 (89.5%) patients presented with deficiency of at least one type of all measured immunoglobulins IgA, M, G, E, and IgG_1-4._

According to major immunoglobulin class division, IgE deficiency was the most common [13/25 (52%)], followed by IgM at 10/25 (40%), IgG at eight of 25 (32%), and IgA at five of 25 (20%). Eight of the 25 patients (32%) had abnormalities in at least two major immunoglobulin (IgG, IgA, IgM) measurements, suggesting CVID-like phenotype.

Six of the patients had selective IgED (24%), three had selective IgAD (12%), and two had selective IgMD (8%). With regard to IgG subclass deficiencies, the most prevalent was IgG4 deficiency at six of 24 (25%), followed by IgG1 at two of 20 (10%) and IgG3 at two of 24 (8.4%).

Patients with 18q del exhibited significantly decreased CD4, Treg FOXP3+, TregFOXP3+Helios+, TemCD4 cell numbers in comparison with the control groups. The control groups consisted of 24 T1DM patients and 28 healthy subjects.

Hypogammaglobulinemia was confirmed in 10/13 patients with the history of severe/recurrent infections. IgED was found in nine of 14 patients with autoimmunity; in five cases, it was selective. All patients with sIgAD had autoimmune phenomena.

### Lymphocyte Subset Phenotyping

Lymphocyte subset phenotyping was feasible in 24 subjects from the 18q del cohort. The controls comprised 24 patients with T1D (autoimmune) diabetes and 28 healthy subjects (**Table 4**).

**Table 4:** The proportion (%) of CD4+ lymphocytes in patients with 18q del, patients with T1D (control group), and 28 healthy controls.

	18q del (*n* = 24)	T1D (*n* = 24)	Control (*n* = 28)	*p*-value in ANOVA
**T subsets (%)**				
**CD4+**				
**Median**	21.82	54.27	40.50	
**Mean (min–max)**	22.87 (4.42–50.59)	56.94 (43.93–78.9)	40.94 (24.07–56.12)	<0.0001
**SD**	11.42	10.47	7.61	
**Tnaive**				
**Median**	62.49	63.02	53.85	
**Mean (min–max)**	58.54 (28.31–82.55)	62.08 (19.88–82.93)	50.08 (11.67–58.68)	<0.0003
**SD**	14.66	15.52	16.68	
**Tcm**				
**Median**	23.82	24.29	30.32	
**Mean (min–max)**	25.37 (12.35–37.6)	22.91 (6.65–39.18)	32.91 (17.00–58.68)	<0.0001
**SD**	7.32	9.25	11.15	
**Tem**				
**Median**	9.8	7.08	13.96	
**Mean (min–max)**	9.8 (2.08–28.4)	8.091 (2.87–18.71)	32.91 (17.00–58.68)	<0.0001
**SD**	6.97	4.261	11.15	
**Treg FOXP3+**				
**Median**	2.2	4.88	6.00	
**Mean (min–max)**	2.91 (0.5–8.96)	5.26 (2.35–9.24)	5.95 (2.30–9.40)	<0.0001
**SD**	2.03	1.73	1.99	
**Treg FOXP3+Helios+**				
**Median**	6.41	73.59	78.54	
**Mean (min–max)**	9.17 (2.86–31.89)	75.27 (36.92–95.39)	78.65 (56.66–97.11)	<0.0001
**SD**	7.616	12.73	11.09	

### Routine Laboratory Parameters and Clinical Data

**Figure 2:**
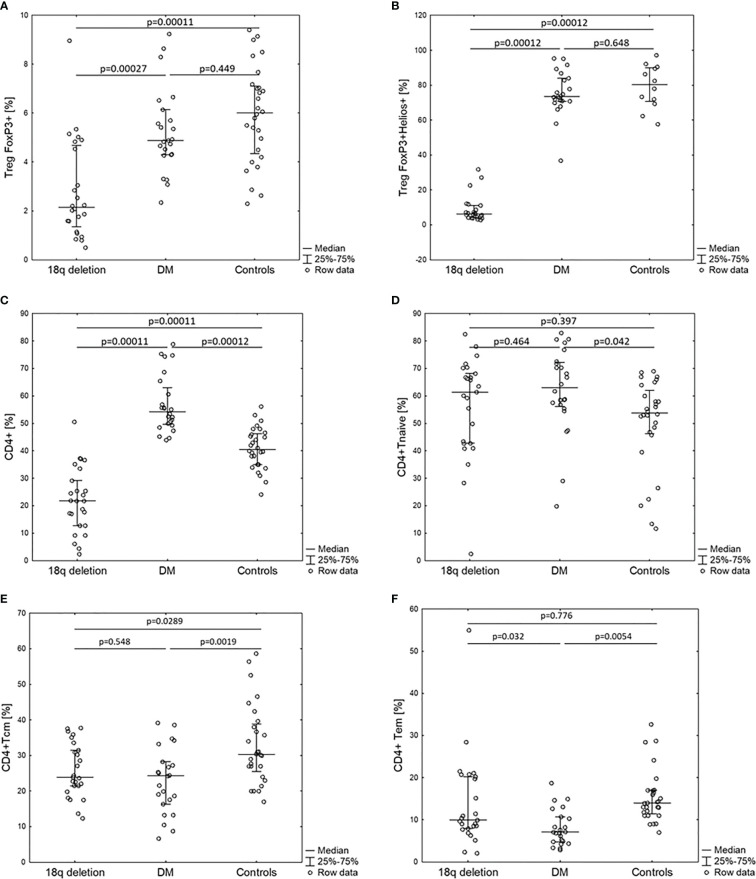
Percentage of CD4+ lymphocyte subpopulations of patients with 18q deletion syndrome, type 1 diabetes (DM), and healthy controls. **(A)** CD4+CD25+FOXP3+, **(B)** CD4+CD25+FOXP3+Helios+, **(C)** CD4+, **(D)** CD4+naïve, **(E)** CD4+T central memory, **(F)** CD4+T effective memory.

**Figure 3:**
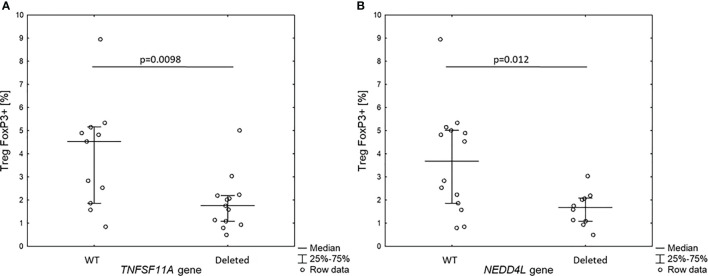
Percentage of Treg FOXP3+ cells in 18q del patients without and with haploinsufficiency of TNFSF11A **(A)** and NEDDL4 genes **(B)**.

Total white blood cell (WBC) counts and lymphocyte counts as well as the CD4/CD8 ratio did not differ among the 18q del, T1DM subjects, and the healthy controls. Patients with 18q del exhibited significantly decreased percentage of CD4+CD25+ FOXP3+Treg and CD4+CD25+FOXP3+Helios+Treg (Helios+ cells originate from the thymus) cell subpopulations in comparison not only with healthy controls but also with T1DM groups (*p* < 0.00011 and *p* < 0.00027, respectively) (**Figure 2A, B** **)**. This was also true for the percentage of total CD4+ lymphocytes (in both cases *p* < 0.00011). Scatter plots depicting the deficiency of CD4+CD25+FOXP3+ and CD4+CD25+FOXP3+ Helios+ lymphocyte subpopulations in a patient with 18q deletion are shown in **Figure 3**. There was no difference in the percentage of CD4-naïve lymphocytes, and CD4 Tem cells between 18q del and the heathy controls (*p* < 0.397 and *p* = 0.776, respectively). The percentage of CD4 Tcm lymphocytes was lower in both the 18q del and the T1DM group than in the healthy controls, *p* = 0.0289, and *p* = 0.0019, respectively, but there was no difference in the percentage of CD4 Tcm lymphocytes between the 18q del and the T1DM group, *p* = 0.548 (**Figure 2E**).

The T1DM group had significantly more CD4+ and CD4-naïve lymphocytes than the healthy controls, *p* < 0.00012 and *p* < 0.042, respectively (**Figure 2C, D** **)**. On the other hand, the percentage of CD4+ Tcm lymphocytes was lower in the T1DM group than in the healthy controls, *p* = 0.0019. The percentage of CD4 Tem lymphocytes was statistically lower than in the controls but also lower than in patients with 18q del (**Figure 2F**)

### Gene Prioritization

After four consecutive iterations, 10 top candidate genes were selected, all of them having similar bioinformatic behavior to the learning set of genes associated with primary immunodeficiency and autoimmunity features (**Supplementary Table S2**).

This included the following genes: *SOCS6*, *BCL2*, *MALT1*, *SMAD4*, *PIK3C3*, *NEDD4L*, *TNFRSF11A*, *NFATC1*, *SMAD2*, and *SMAD7.* Short functional description of each of these genes is included in **Table 5**.

**Table 5:** Known functions of top 10 candidate immunogenes located on 18q.

Top 10 candidates	Function	References
Suppressor of cytokine signaling 6 (*SOCS6*)	SOCS proteins regulate cytokine signals that control the polarization of CD4+ T cells into Th1, Th2, Th17, and T-suppressive FOXP3+ regulatory cell lineages, the maturation of CD8+ T cells from naïve to “stem-cell memory” (Tscm), central memory (Tcm), and effector memory (Tem) states, and the activation of these lymphocytes.	(15)
B-cell lymphoma 2 (*BCL-2*)	*BCL-2* is the founding member of the Bcl-2 family of regulator proteins that regulate cell death (apoptosis), by either inhibiting (anti-apoptotic) or inducing (proapoptotic) apoptosis. It is involved in both immunoglobulin synthesis (mutations result in CVID), lymphocyte development and the establishment of central and peripheral tolerance. *BCL-2* deficiency in mice leads: accelerated lymphoid cell death in thymus and spleen, polycystic kidney, hair hypopigmentation, and distorted small intestine and postnatal growth retardation	(16–18)
Mucosa-associated lymphoid tissue lymphoma translocation gene 1 (*MALT1*)	*MALT1* encoding a caspase-like cysteine protease is a central signaling component in innate and adaptive immunity by regulating NF-κB and other key signaling pathways in different cell types. *MALT1* has a T-cell intrinsic role in regulating the homeostasis and function of thymic and peripheral T cells. Patients with homozygous missense mutation in *MALT1* have severely impaired T-cell proliferation to antigens and antibody responses to vaccination-defective nuclear factor-κB activation and IL-2 production resulting in combined immunodeficiency.	(19–21)
	*MALT1* was found to be crucial for nTreg development. CD4+CD25+FoxP3+ nTreg population was totally absent from *MALT1*−/− thymus, while iTregs can be generated in the absence of *MALT1*.	
	Also, *MALT1* and studies on *MALT−/−* thymus show that it is crucial for thymic CD4+CD25+FoxP3+ nTreg development. Furthermore, patients with homozygous missense mutation in *MALT1* have severely impaired T-cell proliferation to antigens and antibody responses to vaccination.	
SMAD family member 4 (*SMAD4*)	The SMAD4 gene encodes a protein involved in signal transduction of the transforming growth factor-beta superfamily and bone morphogenic proteins by mediating transcriptional activation of target genes. SMAD pathway regulates the production of IgA by B cells.	(22)
Phosphatidylinositol 3-kinase catalytic subunit type 3 (*PIK3C3*)	The lipid kinase *PIK3C3* regulates multiple aspects of endo-membrane trafficking processes. *PIK3C3* is widely expressed by neurons in the CNS. Pik3c3 deficiency results in progressive neuronal degeneration	(23)
	*Pik3c3*-deficient T cells exhibited impaired cellular metabolism, and *Pik3c3*-deficient CD4+ T cells failed to differentiate into T helper 1 cells.	
	*Pik3c3* heterozygous mice are normal and fertile. In contrast, Pik3c3 homozygous mutants are embryonically lethal and die.	
	The *PIK3C3* may be also essential for T-cell development as Pik3c3-deficient CD4+ T cells failed to differentiate into T helper 1 cells.	
Neural precursor cell-expressed developmentally downregulated 4-like (*NEDD4L*)	Neural precursor cell-expressed developmentally downregulated 4-like (NEDD4L), an E3 ubiquitin protein ligase that regulates channel internalization and turnover. It appears to have roles in regulating various respiratory, cardiovascular, renal, and neuronal functions.	(24)
	Nedd4-2 terminates the transforming growth factor β (TGF-β)-induced signal transduction by ubiquitination of linker phosphorylated active Smad2/3.	
TNF receptor superfamily member 11a (*TNFRSF11A*)	*TNFRSF11A* essential for the correct differentiation of medullary thymic epithelial cells (mTECs) and serves as a surface marker of mature mTEC in embryonic and adult thymus. It is also necessary for activation of *AIRE* in the mTECs and therefore for presentation of tissue-restricted proteins by mTecs.	(25–27)
	*TNFRSF11A* is a member of tumor necrosis factor receptor superfamily, expressed mainly in osteoclast precursors, dendritic cells but also in thymic mTECs. It is associated with bone remodeling and repair, immune cell function, and lymph node development. Mutations in *TNFRSF11A* are responsible for familial expansile osteolysis [MIM 174810], expansile skeletal hyperphosphatasia, and early-onset Paget disease. Interestingly, in some patients with osteopetrosis due to mutations in *TNFRSF11A* immunological investigation revealed hypogammaglobulinemia and lack of antibody response to tetanus antigen—the defects are compatible with a diagnosis of common variable immune deficiency (CVID). It is therefore possible that haploinsuficiency of this gene might be responsible for immunoglobulin deficiency in our patients. Moreover, mutations in similar genes *TNFRSF13* (TACI), *TNFRSF13C* (BAFF-R), *TNFRSF12* (TWEAK), and *TNFRSF7* (CD27) cause similar defective immunoglobulin production resulting in CVID or selective IgA deficiency.	
	*TNFRSF11A* mutation is associated with a partial defect in peripheral B-cell maturation, possibly secondary to the absence of the natural site of B-cell development—the bone marrow cavities. It has been also shown that TNFRSF11A−/− mice suffered from a complete absence of peripheral lymph nodes.	
Nuclear factor of activated T cells (*NFATC1*)	*NFATC1* binds CNS1 regulatory region of *FOXP3* gene band and stabilizes FOXP3 expression in Tregs. The decreased *NFATC1* protein expression leads to decreased FOXP3 protein expression in Tregs. The reduced expression of these key Treg transcription factors (*NFATC1* and FOXP3) results in reduced expression of Treg-suppressive cytokines (CD25, IL-10, and TGF-β) and impaired Treg-suppressive function.	(28, 29)
Smad family member2,-4,-7 (*SMAD2,-4,-7*)	The SMAD pathway regulates the production of IgA by B cells, maintains the protective mucosal barrier.	(30, 31)
	Mice deficient in SMAD2 specifically in B cells produce more B cells, but class switching to IgA is reduced. Complementary to the Smad2‐deficient mice, the loss of SMAD7 in B cells resulted in a decreased cellularity of B cells, but the residual B cells exhibited an increased bias for IgA production, which correlated with the peak SMAD2 phosphorylation after TGF‐β stimulation.	
	SMAD pathways regulate cytokine signals that control the polarization of CD4+ T cells into inflammatory T helper-type 17 cells and suppressive FOXP3+ T-regulatory cell lineages.	

**Figure 4:**
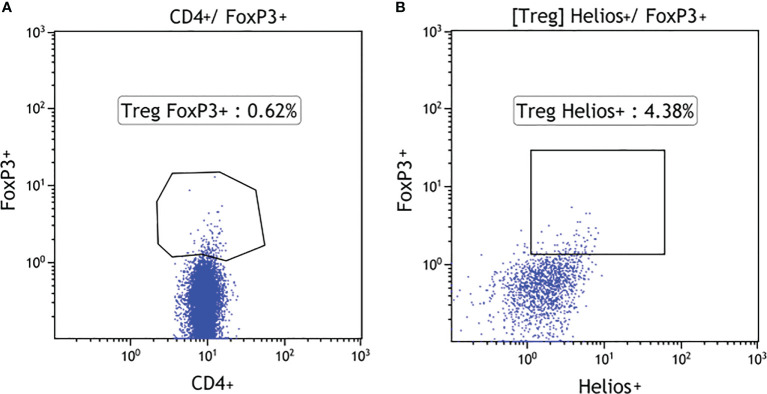
Percentage of Treg FOXP3+ cells in 18q del patients without and with haploin sufficiency of *TNFSF11A* **(A)** and *NEDDL4* genes **(B)**.

Since the patients with 18q deletion syndrome had Treg FOXP3+ deficiency, we analyzed the level of Treg FOXP3+ in the context of gene dose for all 10 genes identified in prioritization approach. Interestingly, we found that patients with haploinsufficiency of *TNFRSF11A* and *NEDD4L* gene had low level of Treg FOXP3+ (1.9 ± 1.0% *vs.* 3.9 ± 12.3%, *p* = 0.0098 and 1.6 ± 0.7% *vs.* 3.8 ± 2.2%, *p* = 0.012, respectively) (**Figure 4**).

## Discussion

The results of the present study confirm our initial assumptions that the 18q deletion is responsible for the increased susceptibility to autoimmune disorders, infections, and allergy in the affected subjects. In this paper, we have also included some novel observations on the pathogenesis of immune dysregulation resulting in this phenotype of 18q deletion syndrome and ring 18.

Firstly, we have demonstrated that, despite relatively young age, more than half of our 18q del cohort suffered from autoimmune disorders. The most common of them were AITDs, followed by alopecia areata, T1DM, and vitiligo. All of these disorders, as well as juvenile rheumatoid arthritis, celiac disease, pernicious anemia, and autoimmune hepatitis, had been previously reported in association with 18q deletions (7, 9, 32–34). In some cases, they coexisted in autoimmune polyendocrine syndromes (6, 35), strongly suggesting impaired central immune tolerance development. Furthermore, we were able to confirm our initial observations (6), that in individuals with 18q del, autoimmunity often coexists with symptoms of immune deficiency and allergy. Nearly half of the cohort had history of severe/recurrent infections and over one-third of the patients reported allergy symptoms.

To the best of our knowledge, only two small studies on 14 and 16 patients aimed to assess the frequency of immune-mediated disorders in 18q del syndrome. Although their results correspond with ours, they should be considered with caution because autoimmune disorders have not been precisely defined and documented (36, 37). Linnankivi reported that three of 14(21.4%) had “autoimmune” disorders (hypothyroidism, iritis), eight of 14 (57%) suffered from frequent respiratory tract infections, and the same percentage had atopic disorders (eczema, food allergy, and asthma) (36).

Cody et al. selected 16 individuals (out of 290) with 18q hemizygosity who had terminal deletion between SERPINB8 and CDH7 that, despite unique breakpoints, encompassed the same set of genes and could be a reference group for distal 18q del. The disorders regarded by the authors as “autoimmune” (myalgia, arthritis, and hypothyroidism) occurred in 31% of subjects and only in females. Atopic diseases were present in 12 out of 16 (75%) of the participants (food allergy in 56%, allergic rhinitis in 50%, eczema in 44%, asthma in 21%) (37).

Secondly, we have shown that individuals with 18q deletion commonly presented with a variable immunoglobulin deficiency, rather than with IgAD alone, which has not been reported so far. Low or absent IgA levels have been regarded as phenotypic feature of 18q del syndrome since 1968 and have been recently estimated to be present in 24%–44% individuals (37–42). In our study, IgAD was present in 20% of our patients (in three of them as sIgAD), but the complex immunoglobulin deficiency mimicking CVID phenotype was far more common. Other types of antibody deficiency, such as selective IgM, IgG_1-4_ subclass deficiency, and IgE deficiency were also frequent (**Figure 1**).

Such alterations in immunoglobulin levels, as well as the progression to CVID (38) or CVID-like disease (immunoglobulin deficiency but good vaccine responses) (35), had been previously mentioned in a few case reports (39, 40). On the other hand, Smith et al. reported two individuals with interstitial deletions encompassing the region 18q21.2-21.3 with anaphylaxis, multiple food allergies, asthma, eczema, immunodeficiency, and extremely high levels of total IgE (43). Another case of persistently elevated IgE concerned a patient with atopic dermatitis and deletion encompassing 18q22.1-18.23 (41). In our study, however, over a half of the 18q del cohort had undetectable IgE levels. Six of them were found to have “selective IgED”—ultra-low IgE levels (<2 kIU/L), with normal levels of other immunoglobulins (13, 42), which was suggested to be a marker of immune dysregulation and autoimmunity (33). Moreover, all 18q del patients with selective IgED had coexisting autoimmune disorder. Unlike hyper-IgE syndrome, which is a well-known form of primary immune deficiency (44), low IgE level is currently not regarded as a clinically important immune deficit. However, it has been recently suggested to be a sensitive indicator for CVID. The variations in immunoglobulin levels point to profound defects in B-cell lineage and immunoglobulin class switching.

Thirdly, we have found that patients with 18q deletions, irrespective of their autoimmune or allergic status, had CD4+CD25+FOXP3+ Treg cell deficiency—the key players controlling reactivity to self-antigens and preventing autoimmunity. Thereby, this group share some striking similarities with the classic examples of the so-called *Tregopathies* like immune dysregulation, polyendocrinopathy, enteropathy, X-linked (IPEX) and IPEX-like monogenic syndromes caused by mutations in *FOXP3* (43), and other genes (*CTLA-4*, *CD25*, *LRBA*, *STAT1*, *STAT3*, *STAT5b*, *BACH2*) mutations, respectively (45).

The profoundly low number of CD4+CD25+FOXP3+Helios+ Tregs (that originate from the thymus) (46), suggests intrinsic dysfunction of thymic maturation of nTregs in individuals with 18q deletions. The patients also exhibited CD4+ and TemCD4+ deficiency which, together with hypogammaglobulinemia, might explain their susceptibility to infections. We therefore suggest that 18q del syndrome should be regarded as another chromosomal syndrome, with compromised immune system, predisposing affected individuals to the development of autoimmune disorders and susceptibility to infections.

We have hypothesized that the observed immune dysregulation results from the deletion of certain genes located on 18q. To address this issue, we performed gene prioritization which indicated “top 10” genes that might be involved in immune system function and central tolerance development. However, only two of them had enough statistical significance for association with FOXP3+Treg deficiency. The first is a neural precursor cell-expressed developmentally downregulated four-like (*NEDD4L*), located on 18q21.31. It is an E3 ubiquitin protein ligase that regulates channel internalization and turnover. It is possibly involved in the termination of the transforming growth factor β (TGFβ)-induced signal transduction by ubiquitination of linker phosphorylated active Smad2/3 (24). Its role in Treg development and function is to be established.

The other gene is called tumor necrosis factor receptor superfamily member 11A (*TNFRSF11A, RANK*), located on 18q21.33. Interestingly, recent studies suggest that *TNFRSF11A* is essential for the correct differentiation of medullary thymic epithelial cells (mTECs) and serves as a surface marker of maturity of these cells in embryonic and adult thymus (25). mTECs play an important role in immune tolerance development by facilitating the clonal deletion of autoreactive T cells, while inducing the generation of thymic T-regulatory cells (47). After activation of transcription factor *AIRE*, they express the whole repertoire of self-antigens and present them to naïve T cells. Mutations in *AIRE* result in promiscuous gene expression in mTECs, leading to autoimmune poliendocrinopathy, candidiasis, ectodermal dystrophy (APECED)—another monogenic syndrome caused by central tolerance breakdown (48). It has been recently shown that *TNFRSF11A* gene product RANK is necessary for activation of *AIRE* (26). Therefore, it is possible that haploinsufficiency of *TNFRSF11A* could be also involved in both CD4+CD25+FOXP3+ cell deficiency.

It is worth mentioning that *TNFRSF11A* (RANK) might also be involved in immunoglobulin synthesis. Mutations of this gene were found in individuals with *osteoclast-poor osteopetrosis associated with hypogammaglobulinemia* (OMIM 612301), manifesting as bone fractures, hematologic abnormalities, recurrent infections, reduced immunoglobulin levels, and lack of antibody response to tetanus antigen (27). In addition, mutations in similar genes, like *TNFRSF13* (TACI), *TNFRSF13C* (BAFF-R), *TNFRSF12* (TWEAK), and *TNFRSF7* (CD27) were found in individuals with selective IgA deficiency or CVID (49, 50). It is, therefore, possible that haploinsufficiency of this gene could explain immune dysregulation with immunoglobulin deficiency in 18q del syndrome.

Although not supported by statistical analysis, the loss of several other immune genes located in 18q might also be responsible for the observed dysregulation in 18q del syndrome (**Table 5**). The spectrum of speculations regarding which gene might be responsible for such phenotype ranges from single gene haploinsufficiency (like in IPEX syndrome) (51), to deletion of a cluster of genes, each gene having small effect, that disrupts gene-gene interactions. The latter scenario might have a greater effect than each of the genes taken separately.

Some practical implications can be seen in our study, such as careful clinical evaluation of patients and supervision since early childhood in terms of higher risk for the immune and endocrine manifestations. In some patients, immunoglobulin substitution may be needed to reduce the frequency of infections (35).

Despite several advantages, such as the highest to date number of involved patients, the novelty of results, as well as the clinical value, our study has some limitations. First of all, the prevalence of the autoimmune disorders, which typically more often appear in adults, might be influenced by the relatively young age of the subjects. Secondly, we have not been able at this point to perform B lymphocyte assessment, as it is done when CVID is suspected, including response to vaccines, which may not necessarily be impaired (35, 52). Also, it might be difficult to find gene(s) involved in immune dysregulation with the approach we used, because the appearance and/or severity of some phenotypic features of the syndrome (e.g., congenital heart defects or narrow ear canals) varies significantly even among family members carrying similar 18q deletion (5, 52). It is thus possible that haploinsufficiency of certain gene(s) predisposing to autoimmunity causes an abnormal phenotype only in the presence of an additional genetic or environmental factors. Since we have not been able to distinguish, which of the top-scoring genes is really associated with the phenotypic features of immunodeficiency in our cohort of patients with 18q deletion, the identification of more patients with different breakpoints within 18q would be helpful to find the responsible gene(s). Moreover, these top 10 genes could be good candidates for future genetic search for mutation among patients with primary immunodeficiency who are negative for mutation in the known PID-responsible genes.

For the purpose of the study, we focused only on some aspects of immunity and further research is needed to precisely describe all possible defects in the innate and adaptive immune system in 18q del syndrome. Taking into account our results and some case reports (35, 53), B-cell immunophenotyping and functional studies on B-cell compartment as well as immunoglobulin class-switching are necessary. Moreover, genomic, transcriptomic, and proteomic data are needed to illicit how genes located on 18q interact with each other and what pathways they create. Also, studies on the influence of knock out of certain 18q genes on the expression of *FOXP3+*, *Helios*, *AIRE*, etc. can provide critical information of Treg cell biology, the role of Treg cell-associated molecules, and the regulation of central and peripheral tolerance in humans.

## Conclusions

Patients with 18q deletions frequently suffer from autoimmune disorders, recurrent infections, and allergy due to immune dysregulation presenting with variable antibody deficiencies and T-regulatory cell deficiency (CD4+CD25+CD127lowFOXP3+). The spectrum of speculations regarding which gene might be responsible for such phenotype ranges from single gene haploin sufficiency to deletion of a cluster of immunogenes located distally to 18q21.

## Data Availability Statement

The datasets presented in this study can be found in online repositories. The names of the repository/repositories and accession number(s) can be found below: the Decipher database (DatabasE of genomiC varIation and Phenotype in Humans using Ensembl Resources; (https://www.deciphergenomics.org/). The accession numbers of the deposited variants in Decipher are 438816–438849.

## Ethics Statement

The studies involving human participants were reviewed and approved by Ethics Committee of Medical University of Lodz, Poland. Written informed consent to participate in this study was provided by the participants’ legal guardian/next of kin.

## Author Contributions

AH and WM contributed to the study design, protocol writing, data collection, analysis, interpretation, and writing and reviewing of the manuscript. MZ, PT, and KW contributed to cell preparation, cell separation, data collection, data analysis and reviewed the report. All other authors contributed to data collection, interpretation and reviewed the manuscript. All authors contributed to the article and approved the submitted version.

## Funding

This work was supported by a Diabetes Poland grant (AH).

## Conflict of Interest

The authors declare that the research was conducted in the absence of any commercial or financial relationships that could be construed as a potential conflict of interest.

## Publisher’s Note

All claims expressed in this article are solely those of the authors and do not necessarily represent those of their affiliated organizations, or those of the publisher, the editors and the reviewers. Any product that may be evaluated in this article, or claim that may be made by its manufacturer, is not guaranteed or endorsed by the publisher.

## Supplementary Material

Supplementary Table 1 Microarray (aCGH) analysis showing cytogenic location and genomic coordinates (GRCh38) of 18 chromosome gains (x3) and losses (x1) of genetic material of the 27 studied subjects.

Supplementary Table 2 The rank of the top 10 candidate genes located on 18q by the ENDEAVOUR prioritization iterations for primary immunodeficiency and autoimmunity.
