# Supplementary material for: Immune Dysregulation in Patients With Chromosome 18q Deletions—Searching for Putative Loci for Autoimmunity and Immunodeficiency
Source: Front Immunol. 2021 Nov 17;12:742834. doi: 10.3389/fimmu.2021.742834 (PMC8637865; doi:10.3389/fimmu.2021.742834)
Supplement: Supplementary Table 1 — Microarray (aCGH) analysis showing cytogenic location and genomic coordinates (GRCh38) of 18 chromosome gains (x3) and losses (x1) of genetic material of the 27 studied subjects. [file Table_1.docx]

Supplementary table 1. Microarray (aCGH) analysis showing cytogenic location and genomic

coordinates (GRCh37) of 18 chromosome gains (x3) and losses (x1) of genetic material of the 27 studied subjects

| **Patient’s No** | **Cytogenic location(genomic coordinates) of 18 chromosome aberration** |
| --- | --- |
| 1 | 46 XX arr 18q12.1-18q21.31(26684735-54370433)x1 |
| 2 | 46 XY arr 18q12.1(29166308-31180985)x1, arr18q21.2(48395091-49401857)x1 |
| 3 | 46 XX arr 18q12.2-18q21.1(34980403-44198268)x1 |
| 4 | 46 XX arr 18q11.2-18q23(22517438-78012829)x1 |
| 5 | 46 XY arr18q21.2-18q23(48581355-78002264)x1 |
| 6 | 46 XY arr 18q21.2-18q23(49902668-78012829)x1 |
| 7 | 46 XY arr 18q21.2-18q23(51285387-78010032)x1 |
| 8 | 46 XX arr 18q21.31-q23(49902668-78012829)x1 |
| 9 | 46 XX arr 18q21.31q23(54563504-78010032)x1 |
| 10 | 46 XY arr 18q21.31-q23(55824970-78012829)x1,12q24.32-q24.33(126901563-133773528)x3 |
| 11 | 46 XY arr 18q21.32-q23(55457282-76805552)x1 |
| 12 | ring 46 XY arr 18p11.32p11.22(139089-8614579)x1, 18q21.32-q23(55949207-76093265)x1 |
| 13 | 46 XY arr 18q21.32-q23(56967282-76113807)x1, 18p11.32(139089-283571)x3 |
| 14 | 46 XX arr 18q21.31-q23 (57426204-78002264)x1 |
| 15 | 46 XY arr 18q21.32-q23(58581355-78002264)x1 |
| 16 | 46 XX arr18q21.32-q23(58660699 - 78012870)x1, 19p13.3(259699-3545499)x3 |
| 17 | 46 XY arr18q21.32-q23(58704982-78010032)x1 |
| 18 | 46 XX arr18q21.33-q23(60154279-78014123)x1 |
| 19 | 46 XX arr 18q21.33-q23(60231849-78010032)x1 |
| 20 | 46 XX arr 18q21.33-18q23(60231849-78010032)x1 |
| 21 | 46 XY arr 18q22.1-q23 (6228124-76805552)x1 |
| 22 | 46 XX arr 18q22.1-q23(64917721-78010032)x1 |
| 23 | 46 XX arr 18q22.2q23 (67650315-78012800)x1 |
| 24 | 46 XX arr18q22.2q23 (67650315-78012800)x1 |
| 25 | ring 46 XY arr 18p11.31p11.32(64.847-3.235.157)x1, 18q22.3-q23(69830202-78010030)x1 |
| 26 | 46 XX arr 18q22.3-q23(70049344-78012829)x1 |
| 27 | ring 46 XY arr 18p11.32p11.23(148963-8434245)x1, 18q23(76724307-78010032)x1 |
