# Supplementary material for: Immune Dysregulation in Patients With Chromosome 18q Deletions—Searching for Putative Loci for Autoimmunity and Immunodeficiency
Source: Front Immunol. 2021 Nov 17;12:742834. doi: 10.3389/fimmu.2021.742834 (PMC8637865; doi:10.3389/fimmu.2021.742834)
Supplement: Supplementary Table 2 — The rank of the top 10 candidate genes located on 18q by the ENDEAVOUR prioritization iterations for primary immunodeficiency and autoimmunity. [file Table_2.docx]

Supplementary table 2. The rank of the top 10 candidate genes located on 18q by the Endeavour prioritization iterations for primary immunodeficiency and autoimmunity.

| **Rank** | **Iteration 1** | **Iteration 2** | **Iteration 3** | **Iteration 4** | **Top 10 candidates** |
| --- | --- | --- | --- | --- | --- |
| 1 | *SOCS6* | *SOCS6* | *SOCS6* | *SOCS6* | *SOCS6* |
| 2 | *BCL2* | *BCL2* | *BCL2* | *BCL2* | *BCL2* |
| 3 | *MALT1* | *TNFRSF11A* | *TNFRSF11A* | *MALT1* | *MALT1* |
| 4 | *NFATC1* | *SMAD4* | *SMAD4* | *PIK3C3* | *SMAD4* |
| 5 | *SMAD2* | *MALT1* | *MALT1* | *SYT4* | *PIK3C3* |
| 6 | *SMAD4* | *NFATC1* | *NFATC1* | *SMAD4* | *NEDD4L* |
| 7 | *TNFRSF11A* | *PIK3C3* | *NEDD4L* | *NEDD4L* | *TNFRSF11A* |
| 8 | *PIK3C3* | *SMAD2* | *PIK3C3* | *PIAS2* | *NFATC1* |
| 9 | *ROCK1* | *NEDD4L* | *SMAD7* | *ROCK1* | *SMAD2* |
| 10 | *NEDD4L* | *SMAD7* | *ROCK1* | *SMAD7* | *SMAD7* |
