# Supplementary material for: Immune Dysregulation in Patients With Chromosome 18q Deletions—Searching for Putative Loci for Autoimmunity and Immunodeficiency
Source: Front Immunol. 2021 Nov 17;12:742834. doi: 10.3389/fimmu.2021.742834 (PMC8637865; doi:10.3389/fimmu.2021.742834)
Supplement: Supplementary file 3 [file DataSheet_1.docx]

ID Decipher of the patients

| No/ Decipher | Chromosome coordinates |
| --- | --- |
| 1      438816 | 46 XX arr 18q12.1-q21.31(26684735-54370433)x1 |
| 2      438817 | 46 XY arr 18q12.1(29166308-31180985)x1, arr18q21.2(48395091-49401857)x1 |
| 3      438819 | 46 XX arr 18q12.2-q21.1(34980403-44198268)x1 |
| 4      438820 | 46 XX arr 18q11.2-q23(22517438-78012829)x1 25% mosaic |
| 5      438821 | 46 XY arr18q21.2-q23(48581355-78002264)x1 |
| 6      438822 | 46 XY arr 18q21.2-q23(49902668-78012829)x1 |
| 7      438823 | 46XY arr  18q21.2-q23(51285387-78010032)x1 |
| 8      438824 | 46 XX arr 18q21.31-q23 (49902668-78012829)x1 |
| 9      438825 | 46 XX arr 18q21.31q23(54563504-78010032)x1 |
| 10    438826 | 46 XY arr 18q21.31-q23(55824970-78012829)1 x,12q24.32-q24.33(126901563-133773528)x3 |
| 11    438827 | 46 XY arr 18q21.32-q23(55457282-76805552)x1 |
| 12    438828 | ring 46 XY arr18q21.32-q23(55949207-76093265)x1, 18p11.32p11.22(139089-8614579)x1 |
| 13    438829 | 46 XY arr 18q21.32-q23(56967282-76113807)x1; 18p11.32(139089-283571)x3 |
| 14    438830 | 46 XX arr 18q21.31-q23 (57426204-78002264)x1 |
| 15    438831 | 46 XY arr 18q21.32-q23(58581355-78002264)x1 |
| 16    438832 | 46 XX arr18q21.32-q23 (58660699 - 78012870)x1, 19p13.3 (259699-3545499)x3 |
| 17    438833 | 46 XY arr18q21.32-q23 (58704982-78010032)x1 |
| 18    438835 | 46 XX arr18q21.33-q23(60154279-78014123)x1 |
| 19    438836 | 46 XX arr 18q21.33-q23(60231849-78010032)x1 |
| 20    438800 | 46 XX arr 18q21.33-18q23(60231849-78010032)x1 |
| 21    438837 | 46 XY arr 18q22.1-q23 (6228124-76805552)x1 |
| 22    438838 | 46 XX arr18q22.1-q23 (64917721-78010032)x1 |
| 23    438845 | 46 XX arr18q22.2q23 (67650315-78012800)x1 |
| 24    438846 | 46 XX arr18q22.2q23 (67650315-78012800)x1 |
| 25    438847 | ring 46 XY arr 18p11.31-p11.32(64847-335157)x1, 18q22.3-q23 (69830202-78010030)x1 |
| 26    438848 | 46 XX arr 18q22.3-q23 (70049344-78012829)x1 |
| 27    438849 | ring 46 XY arr 18p11.32p11.23(148963-8434245)x1, 18q23 (76724307-78010032)x1 |
